# Resolving Sub-Nuclear Architecture from Compartments to Functional Domains

**DOI:** 10.3390/ijms27114680

**Published:** 2026-05-22

**Authors:** Margherita Cavallo, Adel Diaf, Gloria Milanesi, Marco Biggiogera, Claudio Casali

**Affiliations:** Department of Biology and Biotechnology “L. Spallanzani”, University of Pavia, 27100 Pavia, Italy; margherita.cavallo01@universitadipavia.it (M.C.); adel.diaf01@universitadipavia.it (A.D.); gloria.milanesi@unipv.it (G.M.)

**Keywords:** nuclear compartments, chromatin, nuclear bodies, lncRNAs, LLPS, transcription, imaging, super-resolution, electron microscopy, cytochemistry

## Abstract

The cell nucleus is a highly dynamic and complex organelle that orchestrates fundamental cellular processes through its spatial organization. Far from being merely the repository of genetic information, it acts as a regulatory hub whose architecture profoundly influences transcription, RNA maturation and genome maintenance. Dissecting such a multilayered organization requires approaches that integrate molecular profiling with spatially resolved technologies capable of capturing nuclear architecture in situ. In this Review, we discuss classical and emerging imaging strategies that are transforming our understanding of nuclear organization across scales, from multiplexed and super-resolution light microscopy to barcoding-based spatial methods, live-cell imaging, and ultrastructural electron microscopy. Together, these methods are providing crucial insights into the localization and dynamics of RNAs and genomic regions within distinct compartments revealing how nuclear architecture governs genome function.

## 1. Introduction

The eukaryotic nucleus is a highly structured, membrane-bound organelle that harbours the genomic DNA and orchestrates the intricate regulatory programmes that define higher organisms. Elucidating its three-dimensional organization is essential understanding how nuclear structure modulates gene expression with broad implications for developmental biology, neuroscience, and human disease [1,2,3]. Chromatin represents a key structural component of the nucleus, packaging genomic DNA into nucleosomes and higher-order assemblies that are spatially and functionally heterogeneous. Beyond the classical distinction between euchromatin and heterochromatin, the genome is further partitioned into subdomains characterized by distinct epigenetic signatures and regulatory activities [4]. The spatial arrangement of these chromatin states across the nuclear landscape is tightly coupled with transcription and DNA replication, highlighting the need to resolve genome organization in three dimensions. A major advance in the study of nuclear architecture has been the development of high-throughput sequencing (HTS) and chromosome conformation capture (3C) (Hi-C) methods. These allow genome-wide mapping of chromatin interactions revealing key features of higher-order genome organization, including A/B chromatin compartments, which correspond to transcriptionally active (A) and inactive (B) genomic regions, loops and topologically associating domains (TADs) [5,6]. More recent refinements, such as Micro Capture-C ultra (MCCu), have extended this framework to finer scales, allowing the investigation of contacts between regulatory elements and transcription factor binding sites [7]. However, these methods infer spatial proximity from contact frequencies and generally rely on population-averaged measurements. Consequently, complementary approaches are required to directly visualize chromatin architecture in its native three-dimensional context. Imaging-based strategies fulfil this need by allowing the direct observation of chromatin organization within individual nuclei, therefore providing spatially resolved information on domain architecture and cell-to-cell variability [8,9]. More recently, advances in DNA-based Point Accumulation for Imaging in Nanoscale Topography (PAINT), including the development of fluorogenic speed-optimized probes (FSPs), have further improved imaging speed and sensitivity, allowing high-resolution imaging under widefield illumination without the need for optical sectioning [10]. Similarly, improvements in structured illumination microscopy (SIM), such as Lock-in SIM, have enhanced the visualization of cellular structures by reducing background noise leading to a clearer delineation of features such as the perinuclear space [11]. These approaches have also advanced the characterization of nuclear bodies (NBs), whose nanoscale organization and spatial distribution can now be resolved with increasing precision, complementing ultrastructural observations from electron microscopy (EM). For instance, electron spectroscopic imaging (ESI) allowed the characterization of Promyelocytic Leukemia nuclear bodies (PML NBs), revealing a ~250 nm protein-dense core with newly synthesized RNA enriched at their periphery [12,13]. Subsequent methodological advances have provided complementary insight into PML NBs organization, with optimized Lattice SIM^2^ resolving their characteristic ring-like architecture, and cryo-EM providing molecular insight into PML dimerization [14,15]. Together, these observations emphasize the importance of integrating complementary approaches to resolve nuclear organization across scales. However, key aspects of NBs assembly and function remain undefined, particularly for those scaffolded by long non-coding RNAs (lncRNAs), which are difficult to image dynamically in living cells and to resolve across distinct conformational states [16]. Overcoming these challenges will require integrative approaches combining high-resolution imaging with functional and genomic strategies to fully elucidate their roles in nuclear organization and gene regulation.

## 2. Imaging Nuclear Compartmentalization at Different Structural Levels

**Figure 1:**
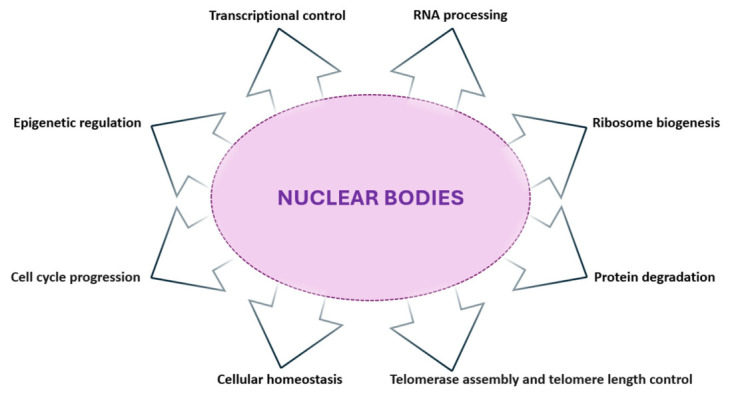
A summary of the diverse cellular and molecular pathways that involve nuclear bodies.

The nucleus is the largest organelle in most eukaryotic cells. Beyond housing the genetic information and serving as the site of DNA and RNA synthesis, transcription and processing, it also has a central role in coordinating cellular functions and architecture [17,18,19]. At larger scales, chromosomes occupy preferential and relatively discrete regions of the interphase nucleus, known as chromosome territories (CTs) [20,21,22]. Complementing this chromatin-based organization, many chromatin-associated factors, together with actively transcribed genes and their RNA products, are further concentrated within specialized nuclear subcompartments known as NBs. These highly dynamic and self-organizing structures change in number, shape and size and their primary role is to concentrate substrates, enzymes and assembly intermediates [23]. By coordinating the spatial organization of regulatory factors and chromatin-associated proteins, NBs function as organizational hubs that facilitate the assembly of molecular components involved in genome regulation [24,25]. Consequently, the dynamic composition and organization of NBs play a crucial role in shaping both local and global chromatin architecture, thereby influencing gene expression profiles in response to diverse cellular states and environmental stresses [26] (Figure 1).

Several distinct nuclear compartments have been characterized, including the nucleolus, PML bodies, Gems, Cajal bodies (CBs), splicing speckles, paraspeckles, Polycomb group bodies, and histone locus bodies (HLBs). Each of these structures contributes to specific aspects of nuclear function, from RNA processing and ribosome biogenesis to transcriptional regulation and epigenetic control [27,28,29,30,31,32]. Despite considerable progress in understanding nuclear organization, many NBs remain the focus of active investigation due to their involvement in a wide range of processes in both physiological and pathological contexts. For instance, Dion et al. [33] recently demonstrated that SON-dependent rehabilitation of nuclear speckles can alleviate proteinopathies, highlighting the functional relevance of these structures in cellular homeostasis. Nevertheless, the mechanisms underlying their biogenesis remain elusive, with ongoing debate regarding the relative contributions of liquid-liquid phase separation (LLPS) and non-coding RNAs (ncRNAs) to their formation and maintenance [34,35,36]. Consequently, the application of high-throughput approaches is essential to systematically investigate their composition, dynamics, and functional roles, ultimately providing deeper insights into this additional layer of gene expression regulation. Traditional transcriptomic approaches such as single-cell RNA sequencing have greatly expanded our understanding of gene expression programmes. However, they require cell dissociation and therefore loss of information about the spatial organization of transcripts within cells and subcellular compartments. Conversely, fluorescence in situ hybridization (FISH) preserves spatial context but allows detection of only a limited number of transcripts because of the small number of distinguishable fluorescent channels. However, a major advance came with the development of multiplexed error-robust fluorescence in situ hybridization (MERFISH) by Chen et al. [37]. This single-molecule imaging approach allows the simultaneous detection and spatial mapping of thousands of RNA species within individual cells. Specifically, each RNA molecule is labelled with encoding probes containing both targeting sequences and readout sequences, which are sequentially detected through multiple rounds of hybridization and imaging. Combinatorial barcoding and error-robust coding schemes allowed accurate identification of transcripts even in the presence of imaging or hybridization noise. This transcriptome-scale spatial profiling approach paved the way for subsequent studies, including the work of Xia et al. [38], who used MERFISH to map the spatial distribution of ~10,000 transcripts and showed that several RNAs preferentially localize to specific subcellular regions, therefore revealing patterns of RNA compartmentalization and cell-cycle-dependent gene expression programmes. Recently, further methodological advances by Takei et al. [39] extended these strategies to the direct visualization of nuclear organization in relation to gene regulation through the development of the two-layer DNA seqFISH+ approach, which markedly increases the multiplexing capacity of imaging-based genomics and allows the spatial mapping of ~100,000 genomic loci in single cells. Using this approach, the authors showed that cell-type-specific regions of heterochromatin marked by histone 3 lysine 27 trimethylation (H3K27me3) and histone 4 lysine 20 trimethylation (H4K20me3) are enriched at specific genes and gene clusters, respectively, and contribute to radial chromosome positioning and inter-chromosomal interactions in neurons and glial cells. They further observed that genomic regions associated with nuclear speckles display characteristic features, including higher GC content, shorter genes, and enrichment of RNAP II Ser5-phosphorylated (RNAP II Ser5-P) and housekeeping genes, indicating an association with constitutively active genomic regions. Moreover, other strategies have coupled multiplexed imaging with super-resolution methods such as PAINT to investigate nuclear compartmentalization at the nanoscale. A recent example is the 2025 study by Rahman et al. who used multiplexed Exchange-PAINT to simultaneously map multiple nuclear proteins and chromatin marks with nanometer precision, showing that active and repressive nuclear environments are organized hierarchically across distinct spatial scales with transcription-associated factors, including RNAP II, p300 and CDK9, not fully co-localizing within the same nuclear domains, but instead forming closely apposed yet spatially distinct nanoscale clusters [40].

## 3. Investigating the Role of lncRNAs in Nuclear Domains Organization

When discussing NBs, it is also important to consider the role of lncRNAs, which were long regarded as non-coding transcripts lacking functional relevance in gene regulation. Extensive work over the past decades has substantially revised this view, revealing that many lncRNAs, also referred to as architectural RNAs (arcRNAs), play fundamental structural roles within the nucleus. They can act as scaffolding components for the assembly of submicron-scale membraneless cellular bodies composed of defined sets of proteins and nucleic acids dedicated to specific molecular functions [41,42,43]. In this context, an important contribution came from the Guttman laboratory, which developed RD-SPRITE to resolve higher-order RNA-DNA interactions and demonstrated that RNAs, particularly lncRNAs, can actively shape nuclear architecture by generating high-concentration territories that recruit regulatory factors and organize essential nuclear functions [44]. However, understanding the conformational dynamics of arcRNAs is essential to provide crucial insights into their role in shaping the nuclear landscape. Berrevoets et al. [45] reconstructed the three-dimensional architecture of paraspeckles defining the spatial organization of the lncRNA Nuclear Paraspeckle Assembly Transcript 1 (NEAT1) through an automated super-resolution workflow combining confocal screening, targeted three-dimensional STED microscopy and quantitative image analysis. Specifically, they uncovered previously unrecognized modes of NEAT1 organization, showing that the 3′ end of NEAT1 adopts a loop-like configuration at the paraspeckle surface, thereby suggesting new ways in which lncRNA conformational dynamics may shape NBs architecture. By contrast, capturing the live dynamics of lncRNAs in relation to nuclear compartmentalization remains substantially more challenging from a microscopy standpoint. Several lncRNAs are predominantly nuclear and tightly associated with chromatin or ribonucleoprotein (RNP) assemblies and therefore occupy crowded environments that are difficult to resolve optically. Additionally, unlike many cytoplasmic mRNAs, lncRNAs often display marked heterogeneity in structure, processing, nuclear retention and turnover, making it difficult to characterize transcription, maturation and degradation processes in living cells [46,47]. Recent advances are beginning to overcome these technical limitations through fluorogenic RNA aptamer-based strategies, in which aptamers render tagged transcripts fluorescent upon binding cognate ligands otherwise non-fluorescent in solution, thus allowing the dynamic visualization of ncRNAs within RNP compartments (for review, see [48]). For instance, Cawte et al. [49] developed Mango II arrays for single-molecule RNA imaging in living cells, improving signal-to-noise ratios and allowing high-contrast visualization of both coding and non-coding transcripts without altering their subcellular localization. Another example includes the work from Vitiello et al. [50] who used Pepper to show that circ-HDGFRP3 dynamically associates with processing bodies (P-bodies) and is recruited into pathological Fused in Sarcoma (FUS) aggregates while nHOTAIRM1, a motor-neuron-specific component of stress granules, could be monitored during the oxidative stress response. Taken together, these studies indicate that, although the live-cell analysis of lncRNAs remains technically demanding, new imaging strategies are opening the way to a more direct understanding of how ncRNAs contribute to the assembly, organization and maintenance of NBs.

## 4. From Activation to Dysregulation: Visualizing Transcription Across Cellular States

Transcription is not just a uniform biochemical reaction, but a temporally and spatially evolving nuclear phenomenon that shapes the nucleus [51,52]. A conceptual timeline can therefore be applied to trace transcriptional activity from its developmental onset during zygotic genome activation (ZGA), through its regulatory reprogramming in differentiated cells, to its eventual perturbation under pathological conditions. Integrating insights from super-resolution microscopy, nuclear compartment biology, and epigenetic regulation highlights how transcriptional dynamics, chromatin topology, and RNA fate are progressively remodelled across the cellular lifespan.

### 4.1. Transcription in the Context of Nuclear Organization

In mammals, transcription begins during ZGA, when control shifts from maternally deposited transcripts to the embryonic genome. Preceding ZGA, the nucleus is predominantly euchromatic and lacks heterochromatic marks [53], a state that permits the passive onset of transcription. Through this nuclear conformation, the spatial positioning of transcribed RNA influences the formation and maintenance of distinct nuclear compartments, including the inactive X chromosome territory, HLBs, nuclear speckles, nucleolar-associated domains, and centromeric silencing regions [44]. As seen in zebrafish embryos, CBs are present from fertilization onwards, while HLBs and nucleolar compartments form foci after a few hours, when histone genes and ribosomal DNA (rDNA) are actively transcribed [54]. The transition to ZGA is accompanied by extensive chromatin remodelling, histone modification reprogramming, and large-scale epigenetic rearrangements [55]. This gene expression shift is driven by pioneer factors, a group of transcription factors with a unique ability to bind target DNA sites that are otherwise inaccessible to the transcription machinery since they are embedded within nucleosomes [56]. In zebrafish embryos, the essential pioneer factor Nanog has been shown to form DNA-bound clusters or “hubs”, particularly at enhancer regions. Using chromatin expansion microscopy (ChromExM), which physically expands the nucleus, improving resolution and reducing molecular crowding, Pownall et al. [57] were able to visualize individual nucleosomes and demonstrate that Nanog organizes into such hubs while recruiting RNAP II Ser5-P at enhancer sites. These hubs bring enhancers and promoters into transient proximity, forming regulatory compartments enriched in transcription factors and coactivators. Within these structures, Nanog and RNAP II occupy distinct spatial domains corresponding to enhancers and promoters, respectively. Following recruitment, RNAP II initiates transcription.

**Figure 2:**
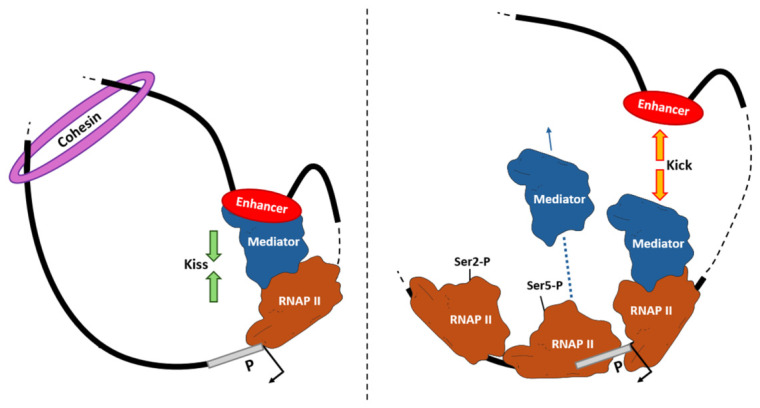
Schematic artwork representing the dynamic “kissing” model. The Mediator complex, a multi-subunit complex conserved in eukaryotes and required for transcription by RNAP II, is composed of four modules: the head, middle, tail, and transiently associated CDK8 kinase modules. It acts as a functional bridge promoting the cohesin-driven enhancer-promoter looping and transcription by RNAP II (phosphorylation of RNAP II Ser5-P is associated with promoter clearance and early transcriptional progression). Mediator can be recruited to transcriptional enhancers through interactions with transcription factors, contacting the transcriptional machinery, and transiently interacting with components of the preinitiation complex (PIC) assembled at core promoters through loss of the kinase module. According to the “kiss and kick” model, transcription itself is responsible for the physical separation of enhancers and promoters, whose contacts are transient and may be released during transcriptional elongation (associated with RNAP II Ser2-P) as the enhancer is “kicked” away from the promoter. P: promoter (modified from [57,60,61,62,63]).

Accumulating evidence suggests that upon transcription initiation, high transcriptional output can contribute to local chromatin reorganization through RNA and protein driven phase separation mechanisms [58]. Although chromatin reorganization establishes a transcriptionally permissive nuclear landscape, productive gene expression ultimately requires the initiation of transcription at individual promoter sites. This process involves the coordinated recruitment of RNAP II, general transcription factors and the Mediator complex to transcription start regions [59]. Because initiation is highly dynamic and transient, dissecting its regulation has posed significant experimental challenges. Cho et al. [60] used fluorescent protein tagging of RNAP II and mediator in mouse embryonic stem cells combined with lattice light-sheet microscopy, a live-cell super-resolution approach that allows visualization of nuclear dynamics with minimal phototoxicity. This strategy allowed the tracking of Mediator and RNAP II, revealing that they assembled into small transient and large stable nuclear clusters. Mediator clusters were frequently associated with enhancer regions and often colocalized with RNAP II clusters. These observations led to the proposal of a dynamic “kissing” model, in which enhancer-bound mediator condensates contact promoter regions transiently to facilitate polymerase recruitment and transcriptional activation across distances exceeding direct enhancer-promoter DNA proximity (Figure 2). Pownall et al. [57] work further complemented this view suggesting that RNAP II forms string-like nanostructures associated with nascent RNA. This process disrupts enhancer–promoter contacts, supporting a refined dynamic “kiss-and-kick” model in which transient interactions enable transcription initiation before being destabilized by elongation. This mechanism provides a structural basis for transcriptional bursting and helps reconcile previously conflicting observations regarding enhancer-promoter interactions.

**Figure 3:**
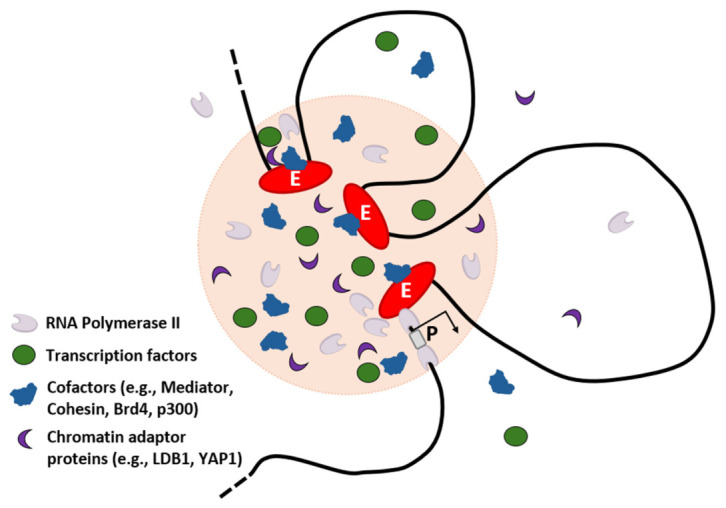
Schematic representation of transcriptional condensates (circle). Transcription factors display propensity to form enriched hubs. These condensates affect various steps of transcription, including transcriptional initiation, pausing, and elongation. Moreover, clustering of super-enhancer has been reported to be regulated by condensates, as well as stages of mRNA maturation, such as the splicing and processing events. E: enhancers; P: promoter (modified from [65,66,67]).

While protein tagging has enabled visualization of transcription condensates and initiation dynamics (Figure 3), the approach relies on overexpression of the tagged protein or genetic modification, both of which can perturb transcription regulation. In order to overcome these limitations, Conic et al. [64] designed a novel approach using fragment antigen-binding (Fab) based probes on live cells following electroporation-mediated delivery into the nucleus. Employing this approach, they simultaneously monitored through live cell imaging histone H3K27 acetylation (H3K27Ac), an epigenetic mark associated with actively transcribed chromatin, and phosphorylation of RNAP II Ser5-P, a modification associated with promoter clearance and early transcriptional progression. Their results revealed that chromatin regions enriched in these activating features are spatially segregated and exhibit diverse temporal dynamics at the nanometer scale. These findings suggest that transcriptional activation does not occur through the stable co-localization of regulatory marks but instead unfolds through dynamically organized and sequential states that reflect distinct stages of gene activation.

Upon transition into productive elongation, RNAP II synthesizes the nascent transcript while progressing along the gene body. A novel technique which allows a direct visualization at the nanoscale resolution of RNA consists in the incorporation of 5-ethynil-uridine (EU) followed by fluorescent labelling. As such, with super-resolution microscopy Castells-Garcia et al. revealed that transcription elongation unfolds within defined chromatin nano-environments. Using stochastic optical reconstruction microscopy (STORM), elongating RNAP II identified by CTD Ser2-P was found to be preferentially enriched at the periphery of nanoscale nucleosome clusters, while densely packed chromatin domains were relatively depleted of polymerase signal. Labelling of nascent RNA further demonstrated that newly synthesized transcripts accumulate within the same nano-domains and extend radially from nucleosome cluster boundaries, thereby creating localized transcriptional microenvironments. Additionally, perturbation of transcriptional activity resulted in measurable changes in nucleosome cluster size and density, indicating that elongation not only responds to chromatin organization but also contributes to its remodelling [68].

After transcription termination, RNAP II is generally released back into the nucleoplasm rather than being recycled, retranscribing the same gene [69]. High-resolution imaging approaches have begun to provide indirect insight into this process. Super-resolution and live-cell imaging studies have revealed that RNAP II forms transient clusters or hubs at active transcription sites, which rapidly dissolve following transcriptional completion [70,71]. However, prior to this event nascent RNA undergoes co-transcriptional splicing, a highly heterogeneous process that does not uniformly affect every intron. The efficiency of splicing is strongly influenced by the spatial organization of the nucleus: transcripts synthesized in close proximity to nuclear speckles exhibit enhanced splicing activity. Using RD-SPRITE Bhat et al. [72] reported a >2-fold higher splicing efficiency for genes transcribed near speckles compared with those located in more distal nuclear regions. The emerging concept of transcription-dependent compartmentalization underscores that RNA synthesis and processing are spatially coordinated within the nucleus. Early EM studies revealed that nascent RNA transcripts and associated RNA-binding proteins accumulate in localized nuclear microenvironments [73]. Similarly, fluorescence imaging has detected persistent RNA signals and localized transcript enrichment after transcription completion [74]. In order to fully comprehend transcription dependent compartmentalization, live correlative light-electron microscopy (CLEM) designed by Haraguchi et al. [75] could provide a bridge by combining the ultrastructural detail of EM with the temporal resolution of fluorescence imaging. This technique combines fluorescent protein tagging with live cell imaging followed by EM, thus allowing the localization of target proteins or even RNA by EU administration in living cells. Once the cellular events of interest occur and are captured using confocal imaging, cells are fixed and processed for EM which enables nanoscale structural resolution.

### 4.2. Transcription Dysregulation in Ageing and Pathology

The dynamic interplay between transcriptional activity and nuclear architecture is a fundamental characteristic of eukaryotic cells. However, the same regulatory architecture that facilitates efficient gene expression in healthy cells can become progressively destabilized under pathological conditions including genotoxic stress [76], ageing [77], and oncogenic transformation [78]. One mechanistic layer underlying these changes involves the regulation of RNAP II Ser5-P associated with transcription initiation and promoter-proximal pausing, while Ser2-P marks productive elongation. In this context, Gyenis et al. [79] reported a global polymerase stalling phenomenon in aged mouse liver. Through EU administration to label RNA, they observed a 1.5-fold reduced RNA signal in old mice and comparable levels of RNAP II Ser5-P, but a significant increase in RNAP II Ser2-P in the aged condition. This indicates impaired transition into productive elongation and suggests that up to 40% of polymerases are stalled, potentially due to accumulated endogenous DNA damage. Notably, ageing-associated transcriptional alterations are highly tissue-specific. For example, global DNA methylation patterns have been shown to increase markedly in mice hepatocytes, whereas more modest changes are observed in mature adipocytes, highlighting the influence of metabolic state and stress exposure on epigenetic remodelling across tissues [80]. Furthermore, ageing is often associated with genomic instability and may ultimately lead to tumorigenesis due to widespread alterations of transcriptional programmes arising from genetic mutations, epigenetic alterations, and disruptions of higher-order chromatin organization. Micronuclei (MN) may arise following mitotic segregation errors resulting in the formation of intact or fragmented chromosomes outside the nucleus. Papathanasiou et al. employed the U2OS 2-6-3 nascent transcription reporter system [81], which incorporates lac operator arrays on chromosome 1 to track locus positioning and an inducible MS2-tagged transcript to visualize nascent RNA, while inducing the formation of MN. Cells forming chromosome 1 MN were assessed revealing that these nuclear architecture aberrations induce persistent transcriptional repression or reduced transcriptional activity of the chromosome. This is accompanied by depletion of active chromatin marks including H3K27ac, decreased RNAP II Ser5-P, and persistence of DNA damage signals such as γH2AX. Notably, these transcriptional defects can persist even after chromosomal reintegration into the primary nucleus, suggesting that architectural perturbations may generate heritable alterations in gene expression states [82].

Ageing-associated alterations extend to the nucleolus, the major site of ribosomal RNA (rRNA) synthesis and ribosome biogenesis. Nucleolar size has been closely linked with ageing and lifespan across multiple organisms, including humans, mice, drosophila, and *C. elegans*, suggesting that this relationship is evolutionarily preserved across taxa. Notably, reduced nucleolar size is associated with increased longevity, while nucleolar enlargement is coupled with cellular decline and senescence [83]. On the contrary, combination of both light and electron microscopy approaches highlighted that perinucleolar heterochromatin is often found enlarged in malignant cells [84]. Recently, several insights were obtained using *S. cerevisiae* as a model. For instance, Carron et al. [85] coupled single molecule localization microscopy (SMLM) and random illumination microscopy to shed light on the organization of nucleolar chromatin, while Gutierrez and Tyler [86] showed that nucleolar enlargement alters condensate properties, allowing invasion of Rad52 and triggering aberrant rDNA recombination. These alterations are accompanied by the progressive loss of cellular homeostasis and replicative senescence characterized by a slow division rate, cell size expansion and dilution of cytoplasmic proteins. However, the relationship between these phenotypes and nucleolar size remains poorly understood, further highlighting the need to elucidate the drivers linking nuclear condensates and cellular homeostasis

Taken together, these observations indicate that pathological states do not simply alter transcriptional output but disrupt its spatial and temporal organization across multiple nuclear scales. From impaired polymerase progression to the destabilization of transcription-associated condensates and nuclear compartments, disease conditions progressively uncouple the coordination between chromatin architecture, transcriptional activity, and RNA processing. As a result, transcription becomes less efficiently compartmentalized, leading to aberrant RNA accumulation, defective processing, and altered gene expression programmes. This integrated perspective highlights that gene expression is governed not only by molecular interactions but also by the physical organization of the nucleus, which both constrains and enables transcriptional activity throughout development, homeostasis, and disease [87,88,89,90].

## 5. Morphological Evidence of Transcription at the Ultrastructural Level

**Figure 4:**
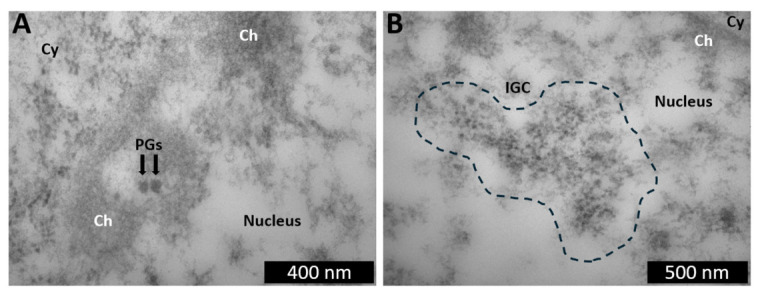
Transmission electron microscopy visualization of (**A**) perichromatin granules (PGs) and (**B**) interchromatin granules (interchromatin granules cluster, IGC) in the cell nucleus of highly transcriptionally active hepatocytes. Ultrathin sections were subjected to EDTA regressive staining to enhance the contrast of RNP particles. Cy: cytoplasm; Ch: condensed chromatin.

One of the major challenges in investigating finely regulated processes such as transcription lies in the intrinsic resolution limits of conventional imaging techniques, which often fail to resolve the structural complexity of subnuclear architecture. Over the past decades, EM has addressed this limitation by providing nanometer-scale resolution, enabling detailed visualization of RNA fibrils and precise subnuclear localization of transcriptional and splicing factors. However, conventional morphological staining methods alone are insufficient to resolve this level of nuclear complexity and must be complemented by precise cytochemical approaches capable of selectively identifying distinct nuclear components. A particularly pioneering contribution in this field was made as early as 1969 by Wilhelm Bernhard, who developed the EDTA regressive staining technique, allowing the selective visualization of RNPs [91,92,93]. This approach relies on the chelation of uranyl ions by neutral EDTA, which preferentially removes uranyl acetate from DNA rather than RNA, thereby enhancing the contrast of RNP-rich structures and enabling the visualization of interchromatin granule clusters (IGCs), considered the morphological counterpart of splicing speckles [94] (Figure 4) and recently shown to be closely associated with chromatin fibers [95], as well as CBs, formerly known as coiled bodies [96], and perichromatin fibrils (PFs) [97], described by Fakan [98] as structures containing nascent and newly synthesized RNA localized at the periphery of the cell nucleus in the perichromatin region (PR). Indeed, high-resolution autoradiography combined with [^3^H]uridine incorporation at different time points revealed the presence of silver grains in close proximity to PFs, both in kidney cells and in rat hepatocytes, where labelling was detectable 5 min after incorporation [99].

**Figure 5:**
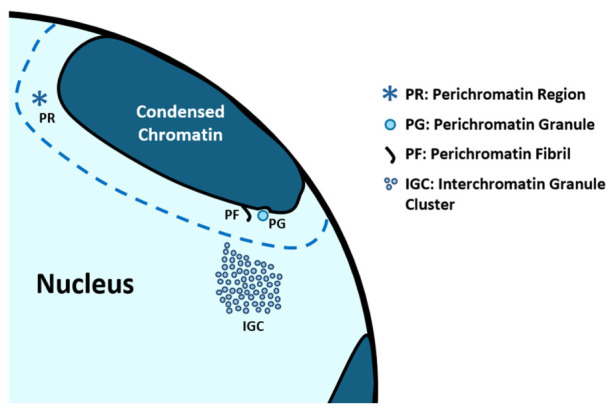
Schematic representation of the PR in the cell nucleus. Originally observed and described by EM, the PR is a ~200 nm-thick area at the periphery of condensed chromatin. The PR is an active and easily accessible nucleoplasmic domain site of multiple molecular events, including RNA transcription and processing, DNA replication and repair. PGs are considered storage of mRNA and are often found in proximity to PFs, which are newly synthesized RNAs and commonly overlap with hnRNPs, snRNPs and the non-snRNP SRSF2 splicing factor. External to the PR, the interchromatin space hosts the IGCs, storage sites for snRNPs and non-snRNPs splicing factors covering a central role in pre-mRNA metabolism [99,100,101].

Overall, these observations reinforced the hypothesis that the PR represents a crucial site of active transcription (Figure 5) (for review, see [100]).

The ultrastructural resolution achieved by TEM further allowed detailed investigation of these domains, revealing the localization of multiple transcription- and processing-related factors. Early evidence supporting the co-transcriptional nature of RNA processing was provided by Fakan et al. [102] who showed that small nuclear ribonucleoproteins (snRNPs) are preferentially associated with perichromatin fibrils, interchromatin granules, and coiled bodies, indicating their early interaction with newly synthesized heterogeneous nuclear RNA. Decades later, Cardinale et al. [103] demonstrated that mammalian cleavage factor I (CFIm), required for the initial step of pre-mRNA 3′-end processing, associates with RNAP II and serine/arginine-rich splicing factor 2 (SC35 or SRSF2) at bromouridine-labelled PFs and IGs-associated zones (IGAZs), providing direct ultrastructural evidence that RNA processing occurs in spatial continuity with transcription. Cmarko and colleagues [104] further reinforced these observations by combining 5-bromo-UTP (BrUTP) microinjection into living cells with immunogold labelling of poly(A) polymerases and heterogeneous nuclear ribonucleoproteins (hnRNPs), which were found to localize at PFs, thereby supporting the concept of co-transcriptional RNA maturation.

For several years, cytochemical approaches such as in situ hybridization and immunocytochemistry coupled with gold particle labelling represented the main strategies for the ultrastructural investigation of transcriptional mechanisms. Nevertheless, staining methods enabling the direct and selective visualization of RNA fibrils remained scarce, as most available procedures depended on the enzymatic removal of DNA from ultrathin sections to render RNA structures detectable. A significant advance was achieved in 1998, when Biggiogera and Fakan introduced a staining procedure employing terbium citrate that enabled direct visualization of RNA fibrils and facilitated the correlation of functional data with their structural organization [99,105]. More recently, the technique has been further refined introducing vapor-based treatments, which enhance contrast and permit a clearer definition of RNA fibrillar architecture as no rinsing steps are required [106].

Despite the valuable insights into transcription provided by cytochemical approaches on ultrathin sections, establishing a direct correlation with functional significance is not always feasible. A crucial example is represented by perichromatin granules (PGs) (see Figure 4), spherical structures detectable upon terbium citrate or EDTA regressive staining, which are widely considered the morphological evidence of mature RNA storage within the nucleus [107]. Ultrastructural and cytochemical studies have shown that PGs are primarily composed of RNA and are closely associated with the final stages of mRNA metabolism. Many experimental observations support the hypothesis that PGs act as transient reservoirs of mature transcripts since their numerical density varies in response to hormonal stimuli that modulate transcriptional activity. For instance, Vazquez-Nin and colleagues showed that ovariectomy reduces both PG abundance and RNA labelling in rat endometrial fibroblasts, whereas estradiol treatment rapidly increases both parameters. These findings led to the proposal that PGs may retain mature RNA until specific signals promote its release [99,108]. Consistent with this interpretation, Bertoni-Freddari et al. [109] reported a decline in PG density with ageing in post-mitotic neurons and hepatocytes, and similar reductions were observed in vitamin-E-deficient mice, further linking PG abundance to transcriptional activity and cellular homeostasis. Despite these observations, assigning a precise function to PGs remains difficult. Their small size precludes biochemical isolation, and unlike other nuclear bodies they are not defined by a known molecular scaffold or key component whose perturbation disrupts their formation, such as NEAT1 for paraspeckles [110]. Addressing this limitation will likely require integrative strategies that combine structural imaging with molecular profiling (e.g., CLIP, eCLIP, RIP-seq). In addition, CLEM, potentially complemented by expansion microscopy (ExM), can bridge fluorescence-based functional information with ultrastructural context.

### Miller Spreads: Ultrastructural Insights into Active Transcription

Ultrastructural studies allowed great improvements in the field of transcription dynamics, relying not only on ultrathin sectioning but also on direct staining approaches performed on nuclear extracts. Indeed, a crucial milestone in this field was the development, in the late 1960s, of the chromatin spreading technique by Oscar Miller and Barbara Beatty, allowing for the first time, the direct visualization of actively transcribed genes [111,112]. Specifically, cells undergo hypotonic lysis, leading to the release and dispersion of chromatin, which is subsequently collected by centrifugation onto EM grids for high-resolution imaging. The resulting images famously revealed the “Christmas-tree” configuration of rDNA transcription units, in which the central axis corresponds to the transcribed rDNA template and the lateral fibrils represent nascent rRNA transcripts [113]. These structures provided compelling ultrastructural evidence that transcription is a highly organized process and, crucially, that ribosome assembly begins co-transcriptionally, as demonstrated by the presence of ribosomal assembly intermediates associated with nascent pre-rRNA. Building upon these fundamental observations, recent advances in cryo-EM have enabled high-resolution structural characterization of such intermediates. For instance, Sanghai et al. [114] recently elucidated the molecular architecture of the large ribosomal subunit (LSU) assembly intermediate that serves as a co-transcriptional checkpoint in yeast, thereby bridging classical morphological observations with a mechanistic understanding of ribosome biogenesis. 

Over the years, the Miller spreading technique has undergone several adaptations, both from a methodological standpoint and in terms of its range of applications. Although Miller and Beatty initially employed phosphotungstic acid (PTA) as the negative stain of choice, the technique has been revisited through the use of alternative staining strategies, such as uranyl acetate and terbium citrate, which have proven particularly suitable in specific experimental contexts [115]. In parallel, what was originally conceived for the study of rDNA transcription was progressively extended, particularly from the 1980s onward, to the investigation of RNAP II-dependent genes, allowing visualization of diverse co-transcriptional processes, including RNP organization, splicing, and 3′-end processing [116,117,118].

## 6. Structural Characterization of the Transcriptional Machinery

The advent of cryo-EM has marked a transformative phase in the evolution of EM approaches, particularly for the structural dissection of in vitro-assembled macromolecular complexes. Importantly, such high-resolution structural information can also provide critical insights into dynamic biological processes. In this context, Pacheco-Fiallos et al. [119] demonstrated that the transcription-export (TREX) complex subunit Aly/REF export factor (ALYREF), long considered primarily an mRNA export adaptor, also plays an active structural role in messenger ribonucleoprotein (mRNP) organization. By integrating complementary approaches, including cryo-EM and tomographic reconstruction, the authors reconstructed the three-dimensional organization of endogenous mRNP particles, showing how TREX complexes engage with the exon-junction complex (EJC) and promote higher-order rearrangements of mRNP architecture. In this context, cryo-EM has been transformative in overcoming previous resolution limitations in the reconstruction of macromolecular assemblies and in elucidating conformational changes within large complexes, including RNAP I [120,121]. Indeed, increasing attention has been directed toward exploiting cryo-EM not only to resolve RNA structures but also to investigate their conformational dynamics. Given that most cellular processes are governed by RNA-protein interactions, understanding the three-dimensional organization and structural plasticity of RNA is essential for gaining mechanistic insights, with broad implications across diverse fields including biomedicine and therapeutic development [122,123,124,125,126].

## 7. Conclusions

Nuclear organization has emerged as a central layer of genome regulation rather than a passive structural backdrop. The combined application of genomic, imaging and ultrastructural approaches has revealed the nucleus as a dynamic and multiscale environment in which chromatin architecture, nuclear compartments and RNA metabolism are functionally intertwined. Despite these advances, key challenges remain, particularly in resolving the dynamic assembly of nuclear compartments and the role of lncRNAs in shaping nuclear architecture. Addressing these questions will require integrative strategies capable of capturing nuclear organization across spatial and temporal scales. In this context, emerging integrative imaging approaches are proving particularly powerful. For instance, recent CRISPR-based RNA imaging platforms, such as CtDeg (CRISPR-dCas13-tDeg), enable target-dependent fluorescence with reduced background, allowing high-specificity visualization of RNA molecules in living cells and providing new insights into lncRNA dynamics, including paraspeckle assembly and NEAT1_2 regulation [127]. Such approaches will be essential to achieve a predictive understanding of genome regulation in both physiological and pathological contexts. At the same time, recent advances combining high-resolution imaging with sequencing-based approaches are reshaping our understanding of nuclear hubs and condensates whose biogenesis is still unclear. These studies reveal that such structures are not merely passive assemblies but can exhibit a highly organized, multiphase architecture, in which RNA molecules act both as structural scaffolds and functional substrates [128]. However, important challenges remain, particularly in capturing their dynamic assembly in living cells in order to identify potential alterations in condensate biogenesis across different physiological and pathological conditions.

## Data Availability

No new data were created or analyzed in this study. Data sharing is not applicable to this article.
